# Postnatal Piezo1 deletion alters collagen fibril architecture in mouse Achilles tendon

**DOI:** 10.1016/j.mbplus.2026.100200

**Published:** 2026-06-17

**Authors:** Ryo Nakamichi, Yuta Fujii, Takahide Matsushima, Merissa Olmer, Martin K. Lotz, Hiroshi Asahara

**Affiliations:** a Department of Molecular and Cellular Biology, The Scripps Research Institute, La Jolla, CA 92037, USA; b Department of Orthopaedic Surgery, Faculty of Medical Development Field, Okayama University, Okayama 700-8558, Japan; c Department of Orthopaedic Surgery, Kyoto Prefectural University of Medicine, Kyoto, Japan; d Department of Systems Biomedicine, Institute of Science Tokyo, Tokyo 152-8550, Japan

**Keywords:** PIEZO1, Tendon, Extracellular matrix, Mechanosensation, Collagen fibril

## Abstract

Tendons are essential connective tissues that transmit mechanical forces from muscles to bones, enabling locomotion and maintaining joint stability. Proper mechanosensation is critical for preserving their extracellular matrix (ECM) integrity. PIEZO1, a mechanosensitive ion channel, has been implicated in regulating tendon architecture under increased mechanical loads, but its role under baseline physiological conditions remains unclear. Here, we generated tendon-targeted Piezo1 conditional knockout (*Piezo1*^*p-t-ko*^) mice using a tamoxifen-inducible *Scx-CreERT2* system. All in vivo experiments were performed in female mice. PIEZO1 deficiency resulted in significantly reduced Achilles tendon thickness and smaller collagen fibril diameters by transmission electron microscopy. RNA sequencing and qPCR analyses demonstrated downregulation of key ECM-related genes, including *Col1a1*, *Dcn* and *Fmod*, as well as the tendon transcription factors *Mkx*. Immunostaining further showed reduced signals of MKX, COL1A1 and DCN. Adjacent muscle morphology and transcriptomes were unaltered, supporting the tendon-selective nature of the observed phenotype. Together, these findings suggest that PIEZO1 contributes to collagen fibril architecture and tendon-associated transcriptional programs in female mouse Achilles tendons under baseline physiological conditions.

## Highlights

• Postnatal Piezo1 deletion reduced Achilles tendon width in female mice.
• Collagen fibril diameters were reduced and fibril packing was altered by TEM.
• RNA-seq showed reduced expression of tendon-associated and ECM-related genes.
• MKX and DCN immunofluorescence signals were reduced in Piezo1-deficient tendons.
• Adjacent skeletal muscle morphology and transcriptome were unaltered.

## Introduction

Tendons serve as crucial mechanical linkages that transmit forces from contracting muscles to bones, thereby orchestrating complex joint motions and sustaining skeletal alignment [1], [2], [3]. They are dense, collagen-rich connective tissues arranged in a hierarchical structure from collagen fibrils to fascicles, and are intimately integrated with muscle and other non-myogenic connective tissue components that contribute to development and homeostasis of the muscle–tendon unit [1], [2], [3]. Because of their constant exposure to mechanical stress, tendons must effectively perceive and adapt to external forces to preserve their structural integrity. Failure to adequately respond to such cues, whether through immobilization, aging, or pathological states, can disrupt this balance, leading to collagen disorganization, and impaired tendon structure [4], [5], [6], [7], [8].

The molecular machinery underlying this mechanosensory capacity has become a focal point of tendon biology. Among the candidates, the mechanically activated ion channel PIEZO1 has emerged as a major conduit by which mechanical stimuli are converted into intracellular calcium signals. Originally characterized in neuronal and vascular contexts, PIEZO1 is now recognized as a versatile mechanosensor across multiple musculoskeletal tissues, including cartilage and bone [9], [10], [11], [12], [13].

Several tendon-associated transcription factors and markers, including Mohawk (Mkx) and Scleraxis (Scx), have been implicated in tendon development, repair, and maintenance of tenocyte phenotype. Although their best-established roles relate to tenogenic lineage specification, tendon maturation, and healing, changes in Mkx- and Scx-associated gene expression can also serve as useful readouts of altered tenocyte transcriptional state in postnatal tendons [14], [15], [16], [17].

Previous studies have demonstrated that PIEZO1 is highly expressed in tenocytes and functions as a critical mechanosensor in tendon biology. Activation of PIEZO1 promotes intracellular calcium influx and stimulates tenogenic transcriptional programs, including Mkx and Scx, thereby promoting ECM synthesis and structural adaptation of tendons [18]. Gain-of-function models of PIEZO1 signaling have been shown to increase tendon thickness, enlarge collagen fibril diameters, and improve elastic energy storage and locomotor performance in mice [18]. In addition, shear stress–mediated activation of PIEZO1 has been reported to regulate tendon stiffness through mechanosensitive calcium signaling [19]. Together, these studies establish PIEZO1 as an important mediator of tendon mechanotransduction.

However, most of the previous work has focused on the effects of enhanced PIEZO1 signaling or mechanical stimulation on tendon adaptation. Whether endogenous PIEZO1 activity is required to maintain tendon extracellular matrix integrity under basal physiological conditions remains unclear.

To address these issues, a tendon-specific Piezo1 conditional knockout mouse model was generated. Using the Achilles tendon as a representative load-bearing tendon, this approach enabled assessment of the contribution of endogenous PIEZO1 to tendon ECM organization under physiological conditions. Postnatal Piezo1 deletion was associated with tendon thinning, reduced collagen fibril diameters, and altered expression of tendon-associated and ECM-related genes in female mice, together supporting a role for PIEZO1 in postnatal tendon collagen fibril architecture and tendon-associated transcriptional programs.

## Results

### Generation of tendon-specific Piezo1 knockout mice

**Fig. 1:**
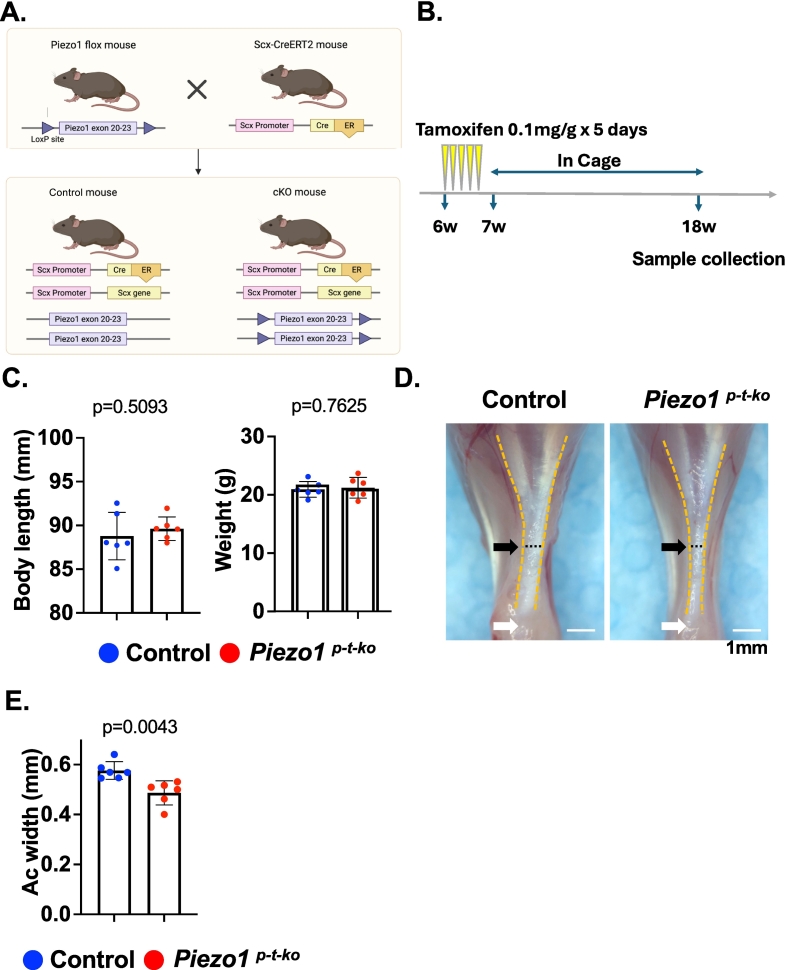
Generation of tendon-specific Piezo1 knockout mice and gross tendon phenotype. **(A)** Breeding scheme for generating tendon-specific Piezo1 knockout female mice (*Scx-CreERT2 (+) Piezo1*^*flox/flox*^ = *Piezo1*^*p-t-ko*^) and Control (*Scx-CreERT2 (+) Piezo1*^*+/+*^ = Control) mice. Created in https://BioRender.com**(B)** Tamoxifen induction protocol. Tamoxifen (100 mg/kg/day, i.p.) was administered for 5 consecutive days starting at 6 weeks of age, and tissues were collected at 18 weeks. **(C)** Body length and body weight at 18 weeks of age in Control and *Piezo1*^*p-t-ko*^ female mice (*n* = 6 per group). **(D)** Representative macroscopic images of Achilles tendons from Control and *Piezo1*^*p-t-ko*^ female mice at 18 weeks. Black arrows indicate the Achilles tendon and white arrows indicate the calcaneus. Yellow dotted lines indicate the outline of the Achilles tendon. Black dotted lines indicate the approximate location of the tendon region used for collagen fibril diameter analysis. Scale bar: 1 mm. **(E)** Quantification of Achilles tendon width in Control and *Piezo1*^*p-t-ko*^ female mice (*n* = 6 per group). Data are mean ± SD. Unpaired two-tailed Student's *t*-test. (For interpretation of the references to colour in this figure legend, the reader is referred to the web version of this article.)

To elucidate the role of PIEZO1 in maintaining tendon integrity, tendon-specific Piezo1 knockout mice (*Piezo1* ^*p-t-ko*^) were generated by crossing *Piezo1* ^*flox/flox*^ mice with *Scx-CreERT2* mice, followed by tamoxifen administration at 6 weeks of age (Fig. 1A and B**)**. Genotyping of genomic DNA from tendon and adjacent muscle confirmed recombination of the floxed Piezo1 allele in tendon but not in adjacent muscle, indicating tissue-restricted recombination in the *Piezo1* ^*p-t-ko*^ model (Supplementary Fig. 1A). In addition, PIEZO1 immunostaining showed reduced PIEZO1 signal in Achilles tendon sections from *Piezo1* ^*p-t-ko*^ mice compared with Control mice at the analyzed age, providing protein-level validation of reduced PIEZO1 expression in the targeted tendon tissue (Supplementary Fig. 1B and C). Body length and weight at 18 weeks were comparable between *Piezo1* ^*p-t-ko*^ and Control, showing that Piezo1 deletion did not affect overall growth (Fig. 1C).

### Postnatal Piezo1 deletion is associated with reduced tendon thickness and altered collagen ultrastructure

**Fig. 2:**
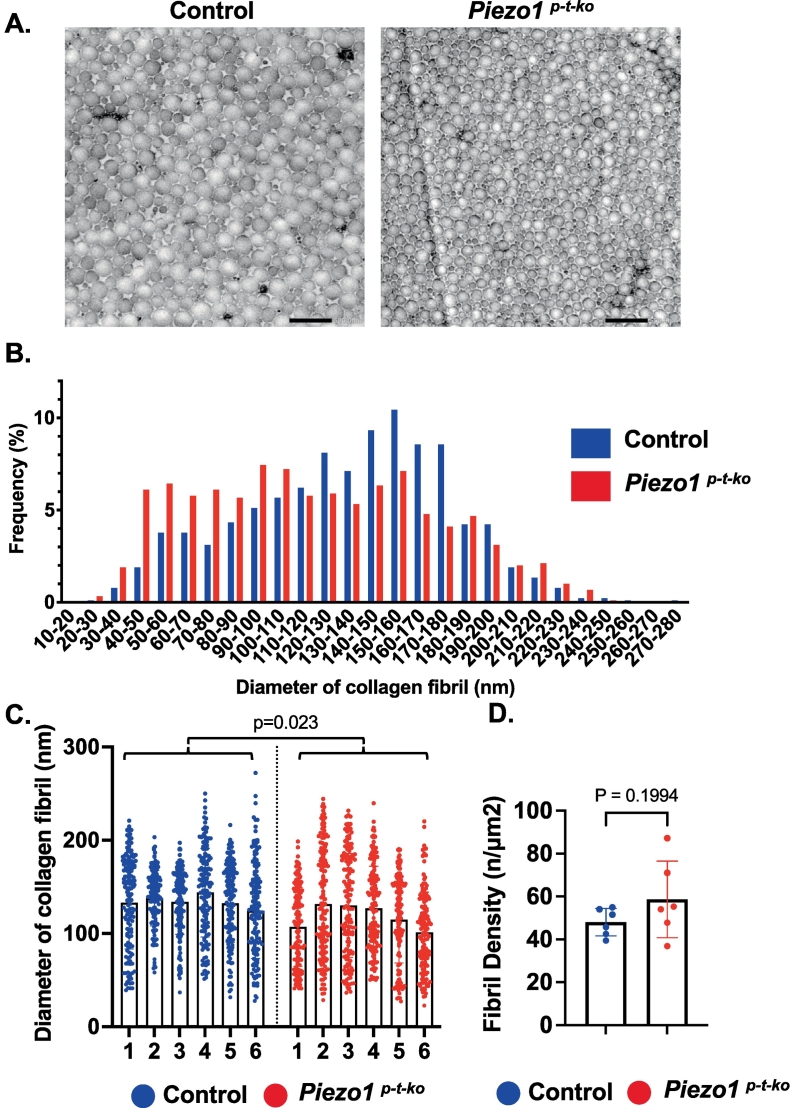
**Postnatal Piezo1 deletion alters collagen fibril ultrastructure in the Achilles tendon**. **(A)** Transmission electron microscopy (TEM) images of mid-substance Achilles tendons from Control and *Piezo1*^*p-t-ko*^ female mice at 18 weeks, showing smaller collagen fibrils in the knockout tendons. Representative transverse views are shown. Scale bars: 500 nm. **(B)** Representative frequency distribution of collagen fibril diameters in Control and *Piezo1*^*p-t-ko*^ female Achilles tendons (n = 6 per group; 150 fibrils analyzed per mouse). **(C)** Mean collagen fibril diameter per mouse in Control and *Piezo1*^*p-t-ko*^ female mice (n = 6 per group). Each small dot represents an individual collagen fibril measurement and bars indicate the mean fibril diameter for each mouse. 150 fibrils were measured per tendon to calculate the per-mouse mean. Statistical analysis was performed using per-mouse mean values. The mean fibril diameter per mouse was significantly reduced in *Piezo1*^*p-t-ko*^ female mice compared with Controls (Control: 134.3 ± 6.4 nm; *Piezo1*^*p-t-ko*^: 118.6 ± 12.8 nm; *p* = 0.023; unpaired Student's *t*-test). (D) Quantification of collagen fibril density in Control and *Piezo1*^*p-t-ko*^ female Achilles tendons. Fibril density was calculated as the number of collagen fibrils per calibrated region of interest area and is expressed as fibrils/μm^2^. Each dot represents the mean fibril density for one mouse. Data are presented as mean ± SD. Unpaired Student's t-test.

Gross inspection revealed visibly thinner Achilles tendons in *Piezo1* ^*p-t-ko*^ mice compared to Control mice (Fig. 1D). Quantitative measurements confirmed a significant decrease in Achilles tendon width in *Piezo1* ^*p-t-ko*^ mice, consistent with macroscopic tendon hypoplasia (Fig. 1E). Transmission electron microscopy (TEM) further demonstrated a reduction in collagen fibril diameters in *Piezo1* ^*p-t-ko*^ female mice, with a leftward shift in the diameter distribution relative to Control (Fig. 2A and B). To align our analysis with previously reported approaches for collagen fibril defects, fibril diameters were measured along the minor axis of each fibril, and diameter distributions were presented as percentage frequency histograms. Quantitative analysis showed a reduction in median fibril diameter (140 nm in Control vs 115 nm in *Piezo1* ^*p-t-ko*^) and a decrease in the first quartile (104 nm vs 77.9 nm), whereas the upper quartile was relatively preserved between groups (165 nm vs 156 nm),indicating an increased proportion of small-diameter fibrils without complete loss of larger fibrils (**Supplementary Table 1**). Consistent with these distributional changes, the mean collagen fibril diameter per mouse was significantly reduced in *Piezo1* ^*p-t-ko*^ female mice compared with Controls (Control: 134.3 ± 6.4 nm; *Piezo1* ^*p-t-ko*^: 118.6 ± 12.8 nm; *p* = 0.023) (Fig. 2C). We further quantified fibril density as the number of collagen fibrils normalized to the ROI area. Fibril density tended to be higher in *Piezo1* ^*p-t-ko*^ tendons compared with Control tendons, although this difference did not reach statistical significance (Fig. 2D). Together, these macroscopic and TEM-based analyses indicate that postnatal Piezo1 deletion is associated with reduced tendon thickness, a shift toward smaller collagen fibrils, and altered fibril packing in female Achilles tendons.

### Transcriptomic and qPCR analyses reveal altered tendon-associated and ECM-related gene expression

**Fig. 3:**
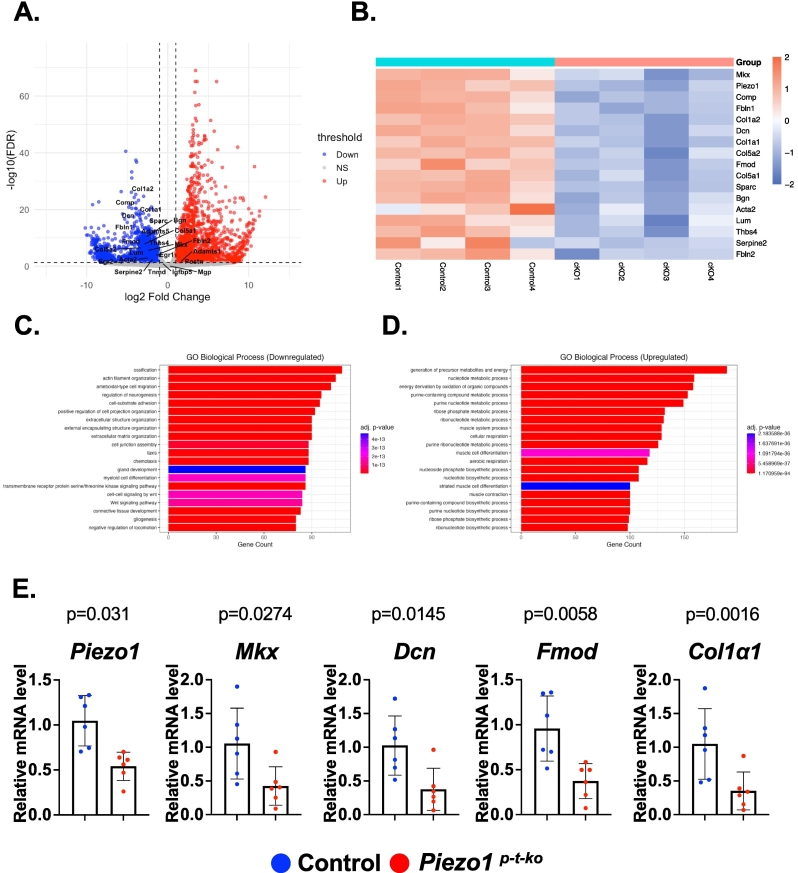
Transcriptomic and qPCR analyses reveal downregulation of tenogenic and ECM genes. **(A)** Volcano plot of differentially expressed genes in Achilles tendons from Control and *Piezo1*^*p-t-ko*^ female mice. Genes in tendon identity and ECM organization are highlighted. *n* = 4 biological replicates per group. **(B)** Heatmap showing expression of selected tendon-associated and ECM-related genes in Control and *Piezo1*^*p-t-ko*^ female Achilles tendons. The colour scale represents relative expression levels shown as z-scores across samples, with red indicating relatively higher expression and blue indicating relatively lower expression. (**C**) GO biological process terms enriched among downregulated genes in *Piezo1*^*p-t-ko*^ tendons compared to controls. (**D**) GO biological process terms enriched among upregulated genes in *Piezo1*^*p-t-ko*^ tendons. **(E)** qPCR validation of RNA-seq findings for *Piezo1*, *Mkx*, *Dcn*, *Fmod* and *Col1a1* in Achilles tendons from Control and *Piezo1*^*p-t-ko*^ female mice (n = 6 per group). Expression levels are normalized to *Gapdh* and presented relative to Control. Data are mean ± SD. Unpaired Student's t-test. (For interpretation of the references to colour in this figure legend, the reader is referred to the web version of this article.)

To explore the molecular underpinnings of these structural changes, bulk RNA sequencing was performed on Achilles tendons from *Piezo1* ^*p-t-ko*^ mice and Control mice. Volcano plot analysis revealed widespread transcriptional alterations in genes associated with tendon structure and function (Fig. 3A). Numerous extracellular matrix–related genes, including *Col1a1*, *Dcn*, and *Fmod*, together with the tenogenic transcription factor *Mkx*, were downregulated in *Piezo1* ^*p-t-ko*^ mice compared to Control mice (Fig. 3B). To provide an unbiased overview of the transcriptional changes associated with postnatal Piezo1 deletion, we performed Gene Ontology enrichment analyses of downregulated and upregulated genes. GO analysis of downregulated genes highlighted biological processes related to skeletal system development and extracellular matrix organization, consistent with the TEM-based structural findings (Fig. 3C). In contrast, upregulated gene sets in *Piezo1* ^*p-t-ko*^ tendons were enriched for actin cytoskeleton-related processes, calcium ion transport, and mitochondrial oxidative phosphorylation pathways, indicating broader non-ECM-related transcriptional changes in this model (Fig. 3D).

To further examine extracellular matrix–related transcriptional changes, we performed a matrisome-focused reanalysis of the RNA-seq dataset [20]. The matrisome volcano plot revealed a consistent reduction of several canonical tendon matrix genes in Piezo1 ^*p-t-ko*^ female tendons, including *Col1a1*, *Dcn*, *Fmod, Comp*, and *Col3a1* (Supplementary Fig. 2A). A heatmap of the top differentially expressed matrisome genes further demonstrated clear segregation between Control and Piezo1 ^*p-t-ko*^ female samples (Supplementary Fig. 2B), supporting the notion that postnatal Piezo1 deletion is associated with altered expression of ECM-associated genes in female Achilles tendons.

Quantitative PCR independently validated the RNA-seq results, showing significant reduction in *Piezo1* and reduced expression of tendon-associated genes, including *Mkx, Dcn, Fmod,* and *Col1a1*, in *Piezo1* ^*p-t-ko*^ mice compared to Control (Fig. 3E). Together, these transcriptomic and qPCR data indicate that postnatal Piezo1 deletion is associated with altered tendon-associated transcriptional programs and reduced expression of selected ECM-related genes in female Achilles tendons.

To further investigate mechanotransduction-related signaling downstream of PIEZO1, we performed gene set enrichment analysis (GSEA) using GO biological processes and MSigDB hallmark gene sets. This analysis revealed enrichment of processes related to actin-mediated cell contraction, actin filament–based movement, and calcium ion transport. In addition, mitochondrial and oxidative phosphorylation pathways were enriched. We next examined the expression of selected genes associated with canonical mechanotransduction pathways. Several genes involved in calcineurin/NFAT signaling (e.g., Rcan1, Rcan2, Ppp3ca, Ppp3cb) and YAP/TAZ signaling (e.g., Yap1, Tead1, Ankrd1) were upregulated, whereas additional pathway components exhibited mixed patterns of upregulation and downregulation. These findings suggest transcriptional remodeling of mechanosensitive pathways in response to Piezo1-deficient tendons, rather than direct evidence of altered pathway activity (Supplementary Fig. 3). In addition, analysis of adjacent skeletal muscle revealed no significant differences in muscle weight or gene expression between genotypes, supporting the tissue-specific nature of the observed tendon phenotype (Supplementary Fig. 4).

### Postnatal Piezo1 deletion is associated with altered selected tenocyte and ECM protein signals

**Fig. 4:**
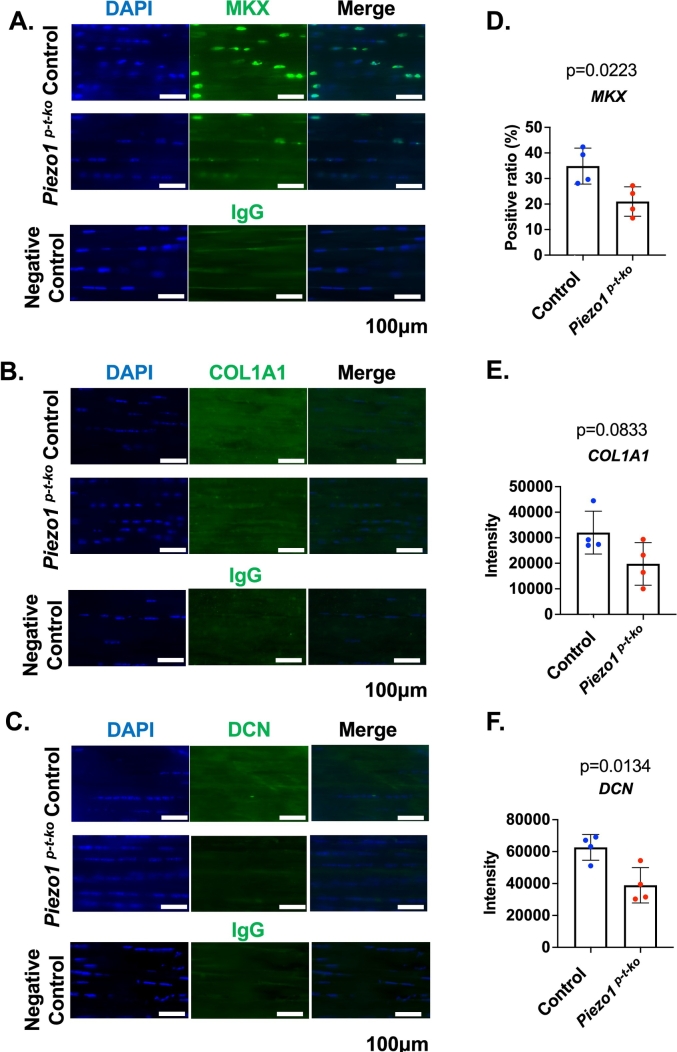
Postnatal Piezo1 deletion reduces MKX and collagen-rich ECM at the protein level. (**A**) Representative immunofluorescence images of Achilles tendon midsubstance sections stained for MKX (green) and counterstained with DAPI (blue) in Control and *Piezo1*^*p-t-ko*^ female mice at 18 weeks. Scale bars: 100 μm. (**B**) Representative immunofluorescence images of COL1A1 (green) and DAPI (blue) in Achilles tendon midsubstance sections from Control and *Piezo1*^*p-t-ko*^ female mice. Scale bars: 100 μm. (**C**) Representative immunofluorescence images of DCN (green) and DAPI (blue) in Achilles tendon midsubstance sections from Control and *Piezo1*^*p-t-ko*^ female mice. Scale bars: 100 μm. (**D—F**) Quantification of immunofluorescence signals. For MKX, the percentage of MKX-positive nuclei relative to total DAPI-positive nuclei was calculated. For COL1A1 and DCN, fluorescence intensity per unit area within the tendon midsubstance region of interest was quantified. Measurements were obtained from three randomly selected fields per tendon and averaged for each mouse (n = 4 mice per group). Data are presented as mean ± SD. Statistical significance was determined using unpaired Student's t-test. IgG negative controls for each staining are shown side-by-side in the main figure and were imaged under the same acquisition settings. (For interpretation of the references to colour in this figure legend, the reader is referred to the web version of this article.)

To examine whether the transcriptional changes were accompanied by alterations in selected tenocyte- and ECM-associated protein signals, immunofluorescence staining was performed on Achilles tendon midsubstance sections. IgG negative controls showed minimal nonspecific background signal under the same imaging conditions, supporting that the observed MKX, COL1A1, and DCN signals were above background (Fig. 4A–C). MKX staining was reduced in *Piezo1* ^*p-t-ko*^ female mice compared with Control female mice, consistent with reduced Mkx transcript expression (Fig. 4A). Quantification of MKX-positive nuclei demonstrated a significant reduction in the proportion of MKX-expressing tenocytes in *Piezo1* ^*p-t-ko*^ female tendons (Fig. 4D). Immunostaining for the extracellular matrix proteins COL1A1 and DCN showed trend toward reduced fluorescence intensity in *Piezo1* ^*p-t-ko*^ tendons compared with Control (Fig. 4B and C), with quantitative image analysis indicating a significant decrease for DCN and a non-significant reduction for COL1A1 (Fig. 4E and F). These selected immunostaining findings are broadly consistent with the RNA-seq and qPCR data showing reduced expression of tendon-associated and ECM-related genes. Together, these findings support an association between postnatal Piezo1 deletion and altered selected tenocyte- and ECM-associated protein signals in female Achilles tendons.

## Discussion

This study demonstrates that PIEZO1-dependent signaling contributes to structural and transcriptional features of postnatal Achilles tendons in female mice under baseline physiological conditions. Using a tamoxifen-inducible *Scx-CreERT2* driver, tendon-specific Piezo1 knockout mice were generated, and loss of PIEZO1 resulted in a visibly thinner Achilles tendon phenotype, reduced collagen fibril diameters, and coordinated downregulation of tenogenic transcription factors and ECM genes. These findings suggest that Piezo1 deletion is associated with altered collagen fibril architecture and tendon-associated transcriptional programs under basal cage activity. Previous work by Passini et al. used *Scx-CreERT2;Piezo1*^*fl/fl*^ mice induced at postnatal days 1–3 and demonstrated that Piezo1 deletion in tenocytes reduces stretch-induced Ca^2+^ responses and decreases the stiffness of tail tendon fascicles in young adult mice [19]. They further showed that elevated PIEZO1 mechanosignaling increased tendon stiffness and strength, whereas collagen fibril size distribution and tissue compactness were not detectably altered in the Piezo1 gain-of-function plantaris tendon [19]. These findings provided important evidence that PIEZO1 regulates tendon mechanosensing and mechanical properties. Building on this work, the present study examined a distinct experimental context: postnatal Piezo1 deletion induced at 6 weeks of age and analysis of load-bearing Achilles tendons at 18 weeks. By inducing Piezo1 deletion at 6 weeks of age and analyzing Achilles tendons in situ, the present study was designed to evaluate the role of PIEZO1 in postnatal tendon structure and tendon-associated transcriptional state beyond early developmental stages while minimizing potential confounding effects of embryonic or early postnatal deletion. Thus, our findings complement the work of Passini et al. by examining collagen fibril architecture, fibril density, tendon width, and tendon-associated transcriptional changes in a distinct tendon and postnatal induction paradigm. Differences between the present findings and those of Passini et al. may be explained by differences in tendon type, genetic model, age at analysis, and experimental endpoint. Passini et al. analyzed tail tendon fascicles and plantaris tendons and found increased stiffness and strength in Piezo1 gain-of-function tendons without detectable changes in plantaris tendon fibril size distribution, implicating collagen crosslinking as a major mechanism [19]. In contrast, a previous gain-of-function study from our group and the present loss-of-function study focused on Achilles tendons, where PIEZO1 activation and deletion were associated with directionally opposite changes in tendon width, collagen fibril diameter, and ECM-related gene expression [18]. These observations suggest that PIEZO1-dependent tendon adaptation may occur at different hierarchical matrix levels depending on anatomical site and loading environment. Because the present study did not include biomechanical testing or collagen crosslink analysis, the observed fibril phenotype should be interpreted primarily as structural evidence of altered matrix maintenance rather than as direct evidence of altered tendon mechanical properties.

Previous gain-of-function studies using a PIEZO1-activating allele suggested that augmented PIEZO1 activity can promote tendon adaptation through a Ca^2+^–calcineurin–NFAT-associated transcriptional program involving Mkx, Scx, and ECM-related genes, accompanied by increased tendon thickness, larger collagen fibrils, and improved elastic energy storage and locomotor performance [18]. In the present loss-of-function model, postnatal Piezo1 deletion was associated with reduced expression of selected tendon-associated and ECM-related genes, reduced immunostaining signals for selected ECM proteins, smaller collagen fibrils, and a thinner Achilles tendon phenotype. Together, these studies suggest that PIEZO1-dependent mechanosensing may influence tendon adaptation at multiple levels, including mechanical properties, collagen fibril architecture, and tendon-associated transcriptional programs. However, in the present study, reduced Mkx and Scx expression should be viewed as part of a broader change in tendon-associated transcriptional state, rather than as evidence that these factors directly mediate the collagen fibril phenotype.

Altered mechanical loading profoundly influences tendon matrix turnover. Experimental unloading reduces collagen synthesis and fibril diameter, whereas increased loading enhances collagen organization and tendon stiffness [6], [7], [21], [22]. One possible interpretation is that Piezo1 deletion may create a state of reduced mechanosensory responsiveness, in which basal mechanical cues are not fully translated into tendon-associated transcriptional responses. The resultant thinning of the Achilles tendon and downregulation of collagen- and SLRP-related genes observed in *Piezo1* ^*p-t-ko*^ mice conceptually parallel the matrix deterioration seen in disuse models, but arise from a cell-intrinsic defect in mechanosensation rather than from reduced external loading per se [7]. These findings suggest that PIEZO1 may contribute to how tenocytes respond to mechanical cues. The present results therefore provide a structural and transcriptional basis for further investigating how altered mechanosensation may contribute to tendon collagen fibril remodeling.

Mechanistically, previous studies have proposed that PIEZO1-dependent Ca^2+^ influx can influence calcineurin–NFAT signaling and tendon-associated transcriptional programs [18]. However, the present data do not establish whether the observed collagen fibril phenotype reflects a direct role of PIEZO1 in fibril assembly or an indirect consequence of altered mechanosensation, downstream transcriptional remodeling, and changes in cell–ECM interactions. In addition, the transcriptomic data do not support a simple linear model in which loss of Piezo1 uniformly suppresses all downstream mechanotransduction-associated pathways. Indeed, several genes associated with calcineurin/NFAT and YAP/TAZ signaling were upregulated in *Piezo1* ^*p-t-ko*^ tendons. One possible interpretation is that these changes represent compensatory or feedback responses to altered mechanosensation and cell–ECM interactions. Disruption of collagen fibril architecture may alter local matrix mechanics, fibril–cell coupling, cytoskeletal tension, or integrin-associated signaling, thereby inducing secondary mechanoadaptive transcriptional programs. In parallel, recent studies have highlighted that mechanical cues activate additional pathways in tendon cells, including FAK, ERK, PI3K/Akt, and YAP/TAZ [23], [24], [25], [26]. In this context, increased expression of selected NFAT- and YAP/TAZ-associated genes may reflect an adaptive response to altered mechanical signaling rather than direct activation of these pathways by PIEZO1. Because the present study assessed mRNA abundance but not NFAT nuclear localization, YAP/TAZ localization, phosphorylation status, or transcriptional activity, these data should be interpreted as pathway-associated transcriptional remodeling rather than definitive evidence of pathway activation. Transcriptomic analysis further revealed enrichment of intracellular metabolic and biosynthetic processes, which may reflect compensatory cellular responses to altered tendon ultrastructure and mechanical signaling [27], [28]. Gene ontology analysis also indicated enrichment of muscle-related processes. While minor contamination from adjacent muscle cannot be completely excluded in Achilles tendon RNA sequencing, such annotations may also arise from cytoskeletal and contractile gene programs activated in tendon cells under mechanical perturbation, suggesting that these transcriptomic changes may reflect mechanoadaptive cellular responses within tendon tissue [29], [30].

An intriguing aspect of this study is the tissue specificity of the phenotype. Despite clear alterations in tendon structure and ECM-related gene expression, *Piezo1* ^*p-t-ko*^ *mice* showed no significant differences in the mass or transcriptional profiles of adjacent skeletal muscles. This suggests that, at least under low-to-moderate loading associated with standard cage activity in this model, tendon-intrinsic PIEZO1 signaling can be perturbed without eliciting overt secondary remodeling in neighboring muscle. Nonetheless, muscles and tendons function as an integrated mechanical unit, and alterations in tendon stiffness or energy storage capacity are expected to influence muscle work and efficiency during dynamic tasks. It will therefore be informative to challenge Piezo1-deficient tendons with controlled exercise paradigms or aging and to quantify how such perturbations reshape muscle–tendon unit mechanics and neuromuscular adaptation.

From a translational perspective, these findings position PIEZO1 as a potential therapeutic entry point for preserving or restoring tendon ECM integrity. Tendinopathies and chronic tendon degeneration are associated with altered mechanical loading, including disuse and overloading, both of which can perturb the balance between matrix synthesis and degradation [31], [32]. Pharmacological or biophysical strategies that modulate PIEZO1 activity or related mechanotransduction pathways may influence tendon-associated transcriptional programs and collagen fibril architecture, although their effects on ECM composition and matrix turnover remain to be determined. In this context, it is noteworthy that low-intensity pulsed ultrasound has been reported to promote osteogenesis in mechanically stimulated periodontal ligament cells via Piezo1 activation, raising the possibility that appropriately dosed mechanical or ultrasound-based interventions could be harnessed to boost PIEZO1-mediated anabolic signaling in tendons as well [33].

This study has several limitations. First, biomechanical properties such as tensile stiffness, failure load, and energy dissipation were not assessed, and behavioral assays of locomotor performance were not performed. Therefore, the conclusions of the present study are restricted to structural and transcriptional endpoints, and no direct conclusions regarding tendon mechanical function can be drawn from the current data. The observed alterations in collagen fibril diameter distribution and fibril density provide structural evidence of tendon ultrastructural changes, but they do not substitute for direct mechanical testing. Future work integrating biomechanical testing will be required to determine whether the observed structural alterations are associated with changes in tendon mechanical properties in vivo. Second, all tendon analyses were performed at a single time point (18 weeks of age, approximately 12 weeks after tamoxifen induction), precluding determination of the onset or temporal progression of tendon thinning and fibril remodeling. Because tamoxifen was administered at 6 weeks of age, the present findings should be interpreted in the context of postnatal tendon maturation and maintenance rather than fully mature adult tendon homeostasis alone. Rescue experiments were also not performed, and the present study cannot establish whether the collagen fibril phenotype reflects a direct effect of Piezo1 deletion on fibril assembly or an indirect consequence of altered mechanosensation, downstream transcriptional remodeling, or changes in cell–ECM interactions. Future studies incorporating longitudinal analyses, later induction time points, rescue experiments, and gain-of-function approaches will be important to define the temporal and causal relationship between Piezo1 deletion and tendon collagen fibril remodeling. Third, the conclusions are based on a single inducible Cre driver and postnatal induction schedule; recombination efficiency and cellular specificity may vary across tendon compartments, developmental stages, or mechanical environments. Piezo1 deletion was supported by genomic recombination PCR, reduced Piezo1 mRNA expression, and reduced PIEZO1 immunostaining signal in Achilles tendon tissue. However, we did not directly assess PIEZO1 channel activity in targeted Achilles tendon cells. Future studies using functional assays, such as Ca^2+^ imaging or electrophysiological analysis of tendon cells, will be required to determine how loss of PIEZO1 alters mechanotransductive channel activity at the cellular level. In addition, Scx-lineage cells can contribute to tendon–bone attachment sites, and the role of PIEZO1 in entheses or other Scx-expressing connective tissues was not examined in this study. Fourth, all in vivo tendon analyses were performed in female mice. Therefore, the present findings should not be generalized to male mice without further validation. Sex hormones have been reported to influence tendon collagen synthesis, extracellular matrix remodeling, and tendon adaptation to mechanical loading, and it remains unknown whether the PIEZO1-dependent phenotype observed here is conserved in male mice or under different endocrine conditions [34], [35], [36]. In addition, the estrous cycle was not synchronized or monitored in this study, and housing-related factors such as group housing and spontaneous cage activity were not independently quantified. Future studies including male mice, controlled hormonal states, and activity monitoring will be required to define the sex- and hormone-dependent aspects of PIEZO1-mediated tendon remodeling. Fifth, mechanotransduction pathway activity, ECM composition, and matrix protein turnover were not directly measured. RNA-seq and qPCR analyses provide information on transcript abundance, and immunostaining provides spatial information for selected proteins such as COL1A1 and DCN, but these approaches do not quantify NFAT or YAP/TAZ activity, global ECM composition, collagen content, crosslinking, or matrix turnover. Therefore, the observed changes in pathway-associated and ECM-related genes should be interpreted as transcriptional remodeling rather than definitive evidence of pathway activation or altered ECM composition. Future studies using pathway-activity assays, proteomics, biochemical collagen assays, crosslink analysis, or metabolic labeling will be required to determine whether the observed transcriptional and immunostaining changes correspond to changes in signaling activity, ECM composition, or protein turnover. Finally, the analyses were restricted to basal cage activity, and the effects of altered loading conditions (e.g., exercise or disuse) on PIEZO1-dependent tendon remodeling remain to be explored.

In conclusion, the present findings support a role for PIEZO1 in tendon-associated gene expression and collagen fibril architecture in female mouse Achilles tendons during postnatal maturation and maintenance. By linking mechanosensitive signaling to tendon-associated transcriptional programs, these findings provide a basis for future studies examining how PIEZO1 influences tendon structure across different developmental stages, loading conditions, and systemic environments. Future studies combining tissue-specific genetic perturbations with controlled mechanical, temporal, and systemic challenges will be essential to define how PIEZO1 and its downstream networks can be targeted to prevent or treat tendon disorders and, more broadly, to support healthy musculoskeletal aging.

## Materials and methods

### Animals and generation of tendon-specific Piezo1 knockout mice

Piezo1 flox mice (*Piezo1*^*flox/flox*^) (B6.Cg-Piezo1^tm2.1 Apat/J, The Jackson Laboratory) were crossed with *Scx-CreERT2* mice (originally generated as described previously and kindly provided by Dr. Ronen Schweitzer) to generate tendon-specific conditional knockout (*Piezo1* ^*p-t-ko*^) mice (Fig. 1A). Tamoxifen (Sigma, T5648) was dissolved in corn oil at 10 mg/mL and administered intraperitoneally at a dose of 100 mg/kg daily for five consecutive days starting at 6 weeks of age to induce Cre-mediated recombination (Fig. 1B and C). Tamoxifen administration at 6 weeks was chosen to induce Piezo1 deletion after the major early phase of tendon development, thereby reducing potential confounding effects of embryonic or early postnatal recombination. This induction schedule was intended to evaluate the role of Piezo1 during postnatal tendon maturation and maintenance, recognizing that tendon collagen fibril maturation and musculoskeletal adaptation can continue beyond this age. *Scx-CreERT2*-positive (Cre+) mice carrying wild-type Piezo1 alleles (*Piezo1*^*+/+*^) and treated with the same tamoxifen regimen were used as Control mice. Controls carried the *Scx-CreERT2* allele to match *Scx* allele dosage between genotypes. Experiments were performed using female mice. Mice were group-housed under specific pathogen-free conditions with a 12 h light/12 h dark cycle and free access to standard chow and water. Mice were sacrificed at 18 weeks of age. All procedures were approved by the Scripps Institutional Animal Care and Use Committee (Protocol # 09–0029-6). Sample sizes were based on prior studies using comparable genetic models and experimental endpoints and are reported in the corresponding figure legends. For tissue analyses, Achilles tendons from the left and right hindlimbs were assigned to different assays to avoid pooling of samples. In the cohort used for ultrastructural analysis, the left Achilles tendon was processed for transmission electron microscopy, while the contralateral tendon was used for histological analyses. In a separate cohort used for transcriptomic analysis, the left Achilles tendon was used for RNA extraction and RNA sequencing, while the contralateral tendon was used for histological analyses. In all experiments, one tendon per mouse was used for each assay.

### Genotyping PCR

Genomic DNA was extracted from Achilles tendons using a DNeasy Blood & Tissue Kit (Qiagen). PCR was performed to detect the floxed and recombined Piezo1 alleles using the following primers:

WT Forward: 5’-CTTGACTGTCCCCTTCCCCATCAAG-3′.

Flox WT Reverse: 5’-CAGTCACTGCTCTTAACCATTGAGCCATCTC-3’.

Flox KO Reverse: 5’-AGGTTGCAGGGTGGCATGGCTCTTTTT 3’.

The expected product sizes were approximately 330 bp for the floxed allele and 230 bp for the recombined knockout allele. PCR conditions were 95 °C for 5 min, followed by 35 cycles of 95 °C for 30 s, 60 °C for 30 s, and 72 °C for 30 s, with a final extension at 72 °C for 5 min. PCR products were visualized on a 2% agarose gel stained with ethidium bromide.

### RNA isolation, library preparation, sequencing, and analysis

Total RNA was extracted from Achilles tendons using the Direct-zol RNA MiniPrep kit (Zymo Research) with on-column DNase I treatment. RNA integrity was assessed using an Agilent 2100 Bioanalyzer. Libraries were prepared from samples with sufficient RNA quality for RamDA-seq library construction.

RNA-seq libraries were generated using the GenNext RamDA-seq Single Cell Kit (TOYOBO). Sequencing was performed on an Illumina NovaSeq X Plus platform to produce 150 bp paired-end reads at an average depth of ∼60 million reads per sample. Quality trimming was conducted with Trim Galore (v0.4.4), and reads were aligned to the *Mus musculus* reference genome (GRCm39) using STAR (v2.7.0f). Gene-level counts were calculated with featureCounts (v1.6.2). Differential expression analysis was performed in R (RStudio environment) using the DESeq2 package. Raw gene-level counts were imported into DESeq2, low-abundance genes were filtered out, and size factors were estimated using the median-of-ratios method. Normalized counts were then modeled with a negative binomial generalized linear model, and Wald tests were used to assess differential expression between groups. *P* values were adjusted for multiple testing using the Benjamini–Hochberg procedure, and genes with a false discovery rate (FDR) < 0.05 were considered significantly differentially expressed. For visualization, variance-stabilizing transformation (VST) was applied to raw count data, followed by row-wise *Z*-score scaling to highlight relative expression differences across samples. Volcano plots highlight genes with an absolute log2 fold change >1 and FDR < 0.05. Gene Ontology enrichment analysis of differentially expressed genes was performed with DAVID 6.8.

Selected transcripts were validated by qPCR. cDNA synthesis was performed with ReverTra Ace (Toyobo), and qPCR was carried out using SYBR Green (Applied Biosystems) on a QuantStudio 5 system. Expression was normalized to *Gapdh* using the ΔΔCt method. *Gapdh* expression was stable across experimental conditions, as confirmed by comparable Ct values among groups, and was therefore used as the reference gene. Primer sequences are provided in **Supplementary Table 2**.

### Histological analysis and immunohistochemistry

Achilles tendons were harvested following euthanasia in accordance with approved institutional protocols, fixed in Z-fix solution (Anatech) for 24 h, and decalcified in TBD-2 solution (Epredia). Tissues were cryoprotected in 30% sucrose, embedded in OCT compound (4583, Sakura Finetek), and frozen in chilled isopentane over dry ice. Serial 8 μm sections were cut on a cryostat.

Immunofluorescence staining was performed after blocking in 2.5% normal horse serum (S-2012, Vector) for 1 h at room temperature. Sections were incubated overnight at 4 °C with primary antibodies: anti-PIEZO1 (1:500, 28511–1-AP, Proteintech), anti-MKX (1:500, LC-B8063, LS Bio), anti-DCN (1:200, BS6582, Bioworld Technology, Inc.), and anti-COL1A1 (1:200, ab21286, Abcam). Alexa Fluor 488- or 647-conjugated secondary antibodies (A-11034, A-31573, and A-21247; Invitrogen) were applied for 1 h, followed by Hoechst counterstaining. For negative controls, isotype-matched IgG was substituted for the primary antibody at the same concentration. Images were acquired using a Keyence BZX700 microscope. All images were acquired using identical exposure and gain settings within each experiment. Quantification was performed in three random fields per section by two blinded investigators using ImageJ. Fluorescence intensity was calculated as mean signal intensity per unit area after background subtraction.

### Transmission electron microscopy

For ultrastructural evaluation, Achilles tendons were fixed overnight in 2.5% glutaraldehyde and 4% paraformaldehyde in 0.1 M sodium cacodylate buffer (pH 7.4), post-fixed in 1% osmium tetroxide and 1.5% potassium ferrocyanide for 1 h at 4 °C, and stained en bloc with 0.5% uranyl acetate overnight. Samples were dehydrated through graded ethanol and acetone, embedded in LX-112 epoxy resin (Ladd), and polymerized. Ultrathin sections were examined at 80 kV with a Thermo Fisher Talos L120C TEM. Images were captured using a CETA 16 M CMOS camera. For each mouse, one Achilles tendon was analyzed, and collagen fibril diameters were measured from transverse sections obtained from the mid-substance of the tendon. 150 fibrils were quantified per tendon across three randomly selected regions using ImageJ by two blinded observers. Individual fibrils were manually fitted with ellipses, and the minor axis (short diameter) of each ellipse was used as the fibril diameter after calibration with the TEM scale bar. In total, approximately 900 fibrils per genotype (6 mice per group) were analyzed. Diameter distributions were presented as percentage frequency histograms using 10-nm bins. In addition to calculating the mean fibril diameter per tendon, fibril diameter distributions were characterized by quartiles and variance to assess changes in fibril size distribution. Fibril density was calculated by counting the number of fibrils within each defined ROI and normalizing this number to the calibrated ROI area. Fibril density is reported as fibrils per μm^2^. Statistical comparisons of mean fibril diameter and fibril density were performed using per-mouse values as biological replicates.

### Statistical analysis

Data are expressed as mean ± SD, and individual data points are shown in all graphs. The statistical test used, exact *p* values, and sample size (n) for each experiment are reported in the corresponding figure legends. Comparisons between two groups were made using unpaired Student's *t-*tests, unless otherwise indicated. All analyses were conducted using GraphPad Prism 9 (GraphPad Software).

## CRediT authorship contribution statement

**Ryo Nakamichi:** Writing – review & editing, Writing – original draft, Visualization, Validation, Methodology, Funding acquisition, Formal analysis, Data curation. **Yuta Fujii:** Writing – original draft, Visualization, Validation, Software, Methodology, Formal analysis, Data curation. **Takahide Matsushima:** Software, Formal analysis. **Merissa Olmer:** Data curation. **Martin K. Lotz:** Writing – review & editing, Supervision, Investigation. **Hiroshi Asahara:** Writing – review & editing, Supervision, Investigation, Funding acquisition, Conceptualization.

## Funding

This work was supported by JSPS KAKENHI (Grant Numbers, JP20H05696), MEXT KAKENHI (Grant Number JP22H05182), 10.13039/100009619AMED (Grant Numbers JP22gm0010009, JP24jf0126010, JP22ama121045, JP24gm2010002, JP25ek0109836), JST Program for co-creating startup ecosystem (Grant Number JPMJSF2313), and NIH (Grant Number R01AR080127) to H.A. and by JSPS KAKENHI (Grant Numbers JP23K08631) and JST FOREST Program (Grant Number JPMJFR220Y) to R.N.

## Declaration of competing interest

The authors declare the following financial interests/personal relationships which may be considered as potential competing interests: Hiroshi Asahara reports financial support and equipment, drugs, or supplies were provided by JSPS, AMED, JST, NIH. Ryo Nakamichi reports financial support and equipment, drugs, or supplies were provided by JSPS, JST. If there are other authors, they declare that they have no known competing financial interests or personal relationships that could have appeared to influence the work reported in this paper.

## Data Availability

Data will be made available on request.

## Appendix A Supplementary data

Supplementary Figure 1. Validation of Piezo1 recombination in Scx-lineage tendons.

Supplementary Figure 2. Matrisome-focused transcriptomic analysis of Control and Piezo1 p-t-ko female tendons

Supplementary Figure 3. Transcriptomic changes in mechanotransduction-related pathways following Piezo1 deletion.

Supplementary Figure 4. Tendon-specific Piezo1 deletion does not alter adjacent muscle morphology or gene expression.

Supplementary figure legends and Supplementary Tables 1 and 2. Legends for Supplementary Figures 1–4, collagen fibril diameter distribution, and primer sequences used for quantitative PCR.
