# Supplementary figures and images for: Postnatal Piezo1 deletion alters collagen fibril architecture in mouse Achilles tendon
Source: Matrix Biol Plus. 2026 Jun 17;31:100200. doi: 10.1016/j.mbplus.2026.100200 (PMC13316760; doi:10.1016/j.mbplus.2026.100200)

Supplementary Figure 1

A.

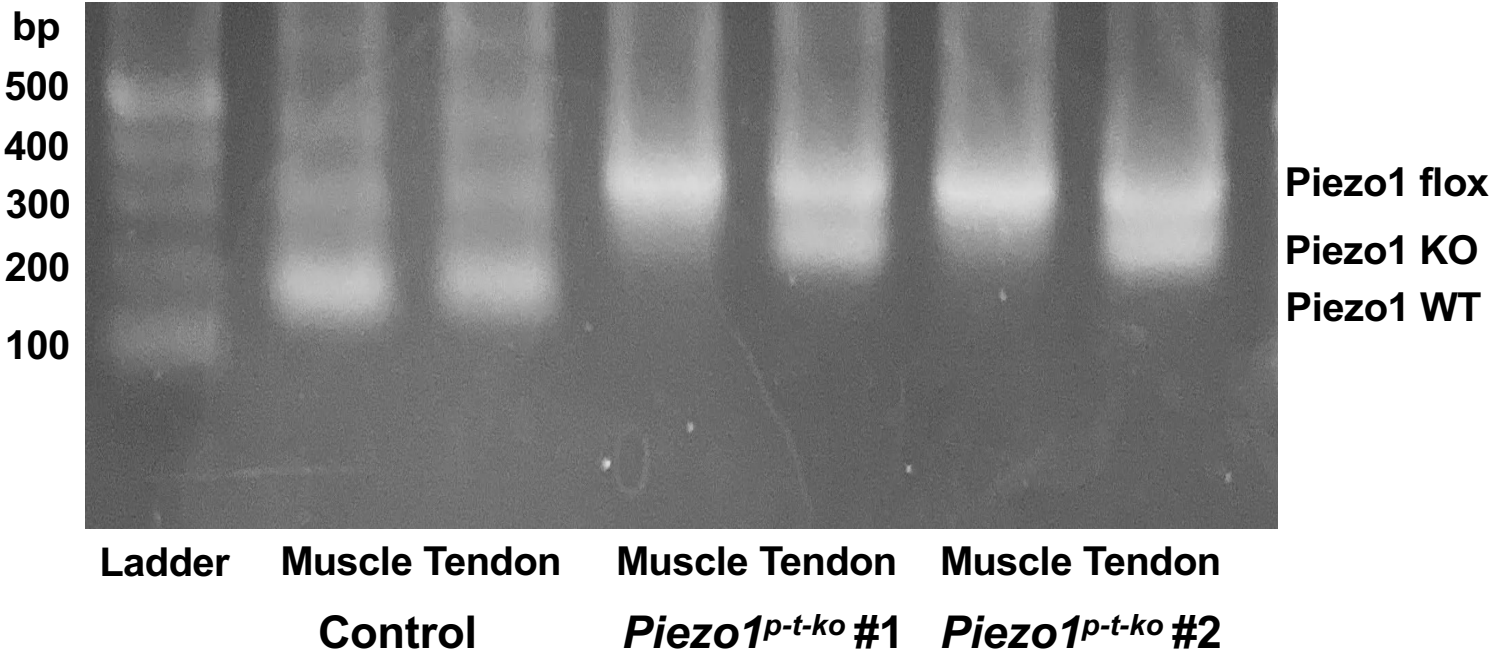

B.

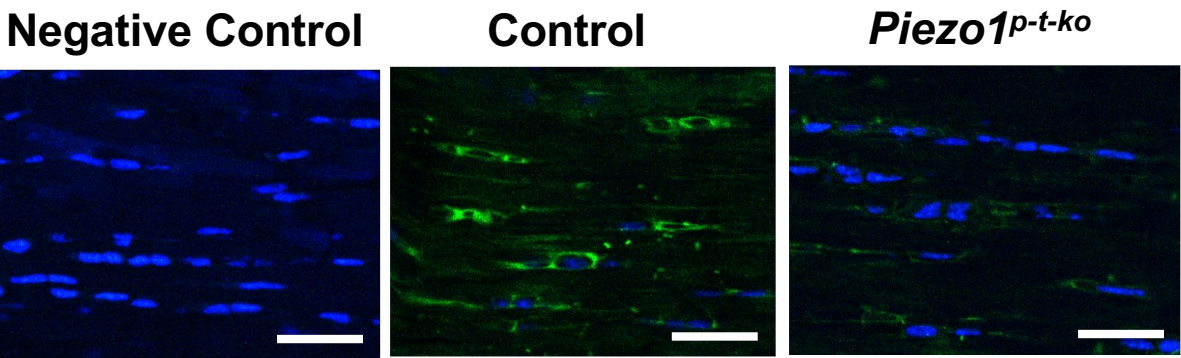

C.

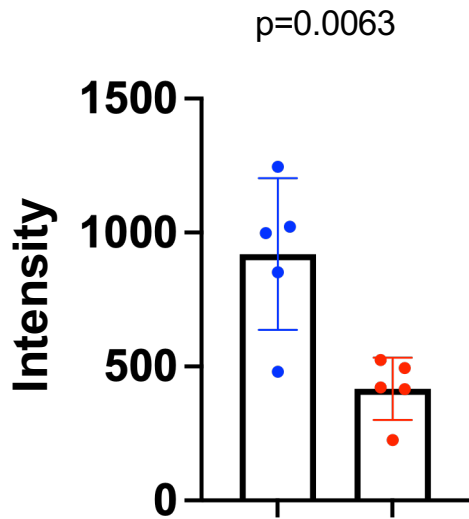

Supplement: Supplementary file 1 — Supplementary Figure 1. Validation of Piezo1 recombination in Scx-lineage tendons. [file mmc1.pdf]

# Supplementary Figure 2

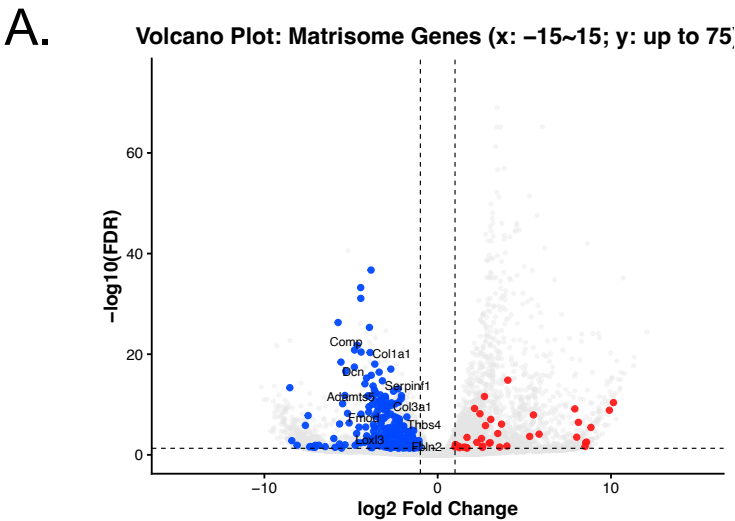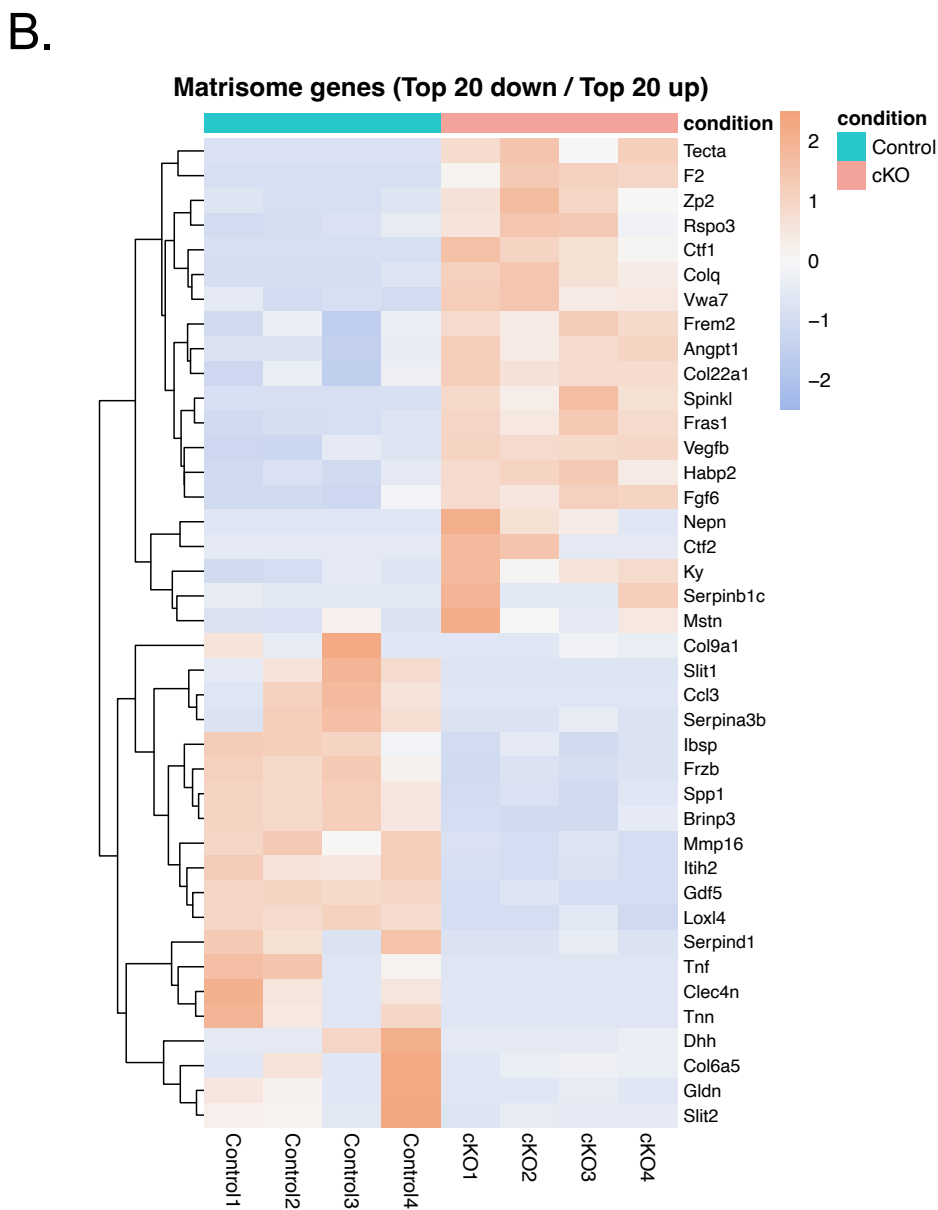

Supplement: Supplementary file 2 — Supplementary Figure 2. Matrisome-focused transcriptomic analysis of Control and Piezo1 p-t-ko female tendons [file mmc2.pdf]

# Supplementary Figure 3

A.

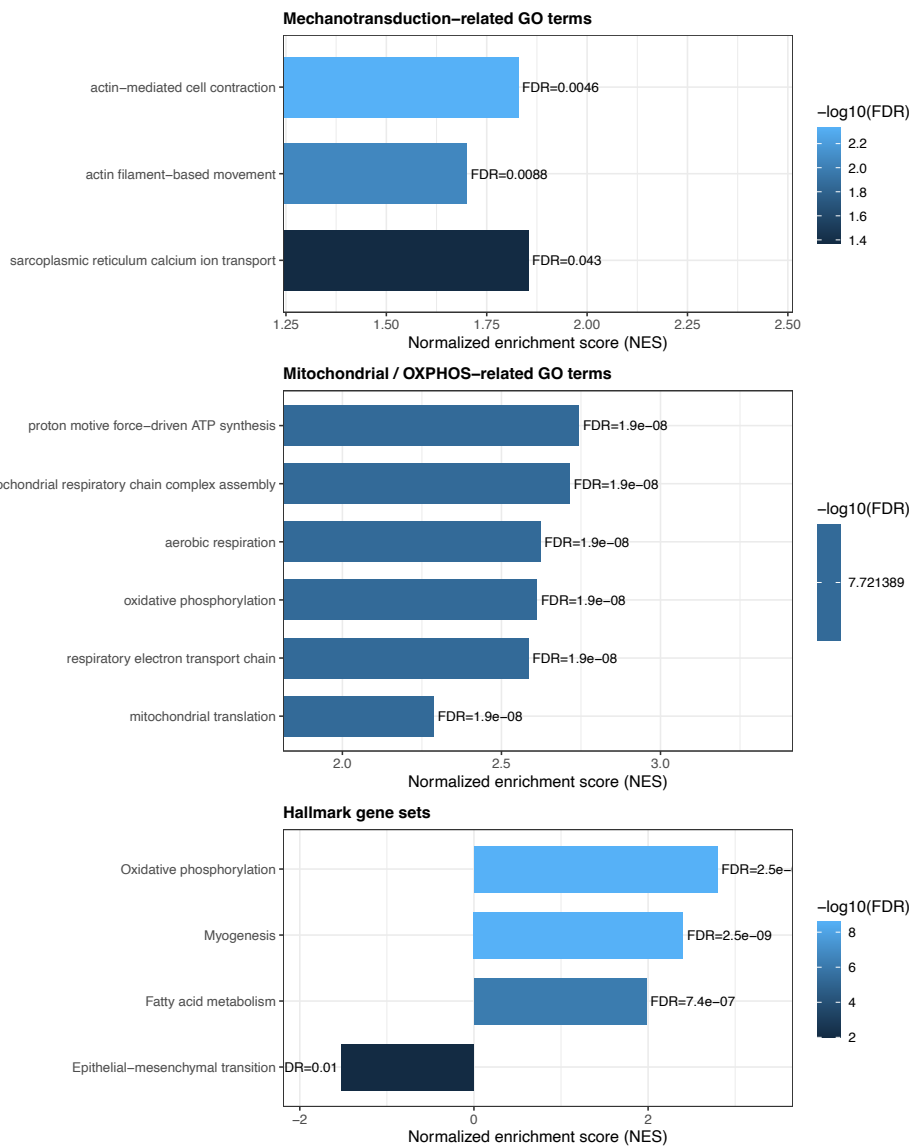

B.

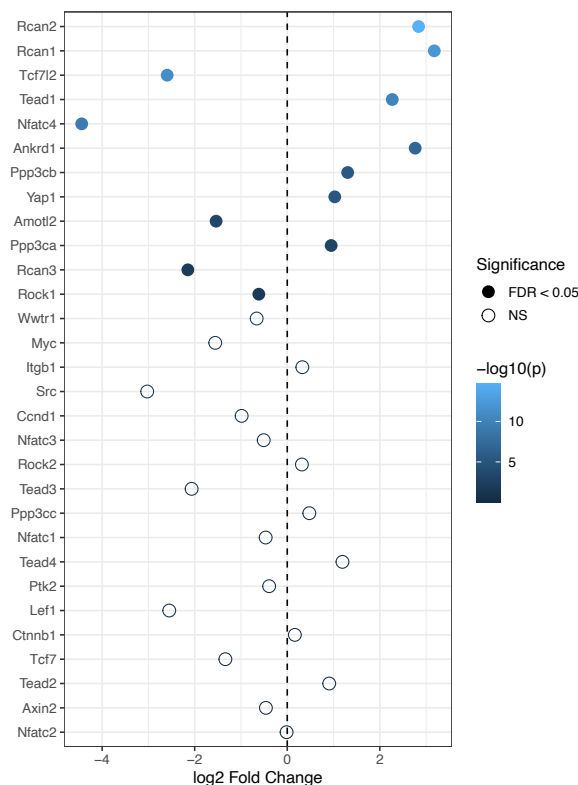

Supplement: Supplementary file 3 — Supplementary Figure 3. Transcriptomic changes in mechanotransduction-related pathways following Piezo1 deletion. [file mmc3.pdf]

# Supplementary Figure 4

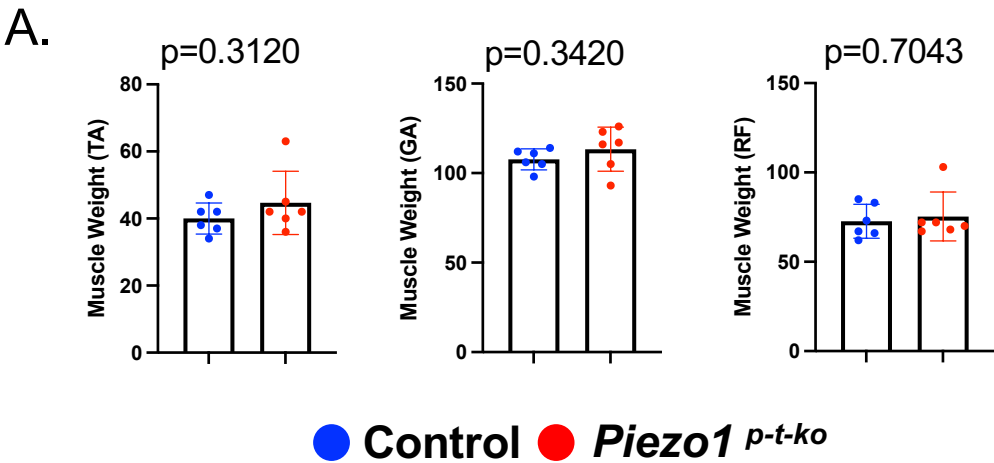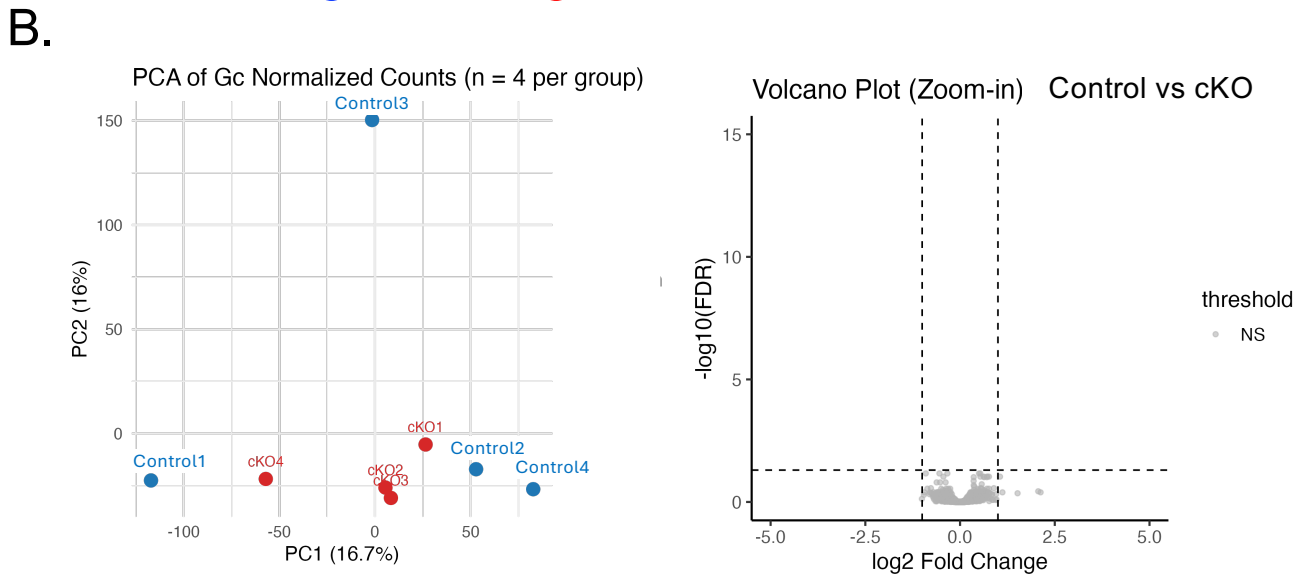

Supplement: Supplementary file 4 — Supplementary Figure 4. Tendon-specific Piezo1 deletion does not alter adjacent muscle morphology or gene expression. [file mmc4.pdf]
