# Supplementary material for: Postnatal Piezo1 deletion alters collagen fibril architecture in mouse Achilles tendon
Source: Matrix Biol Plus. 2026 Jun 17;31:100200. doi: 10.1016/j.mbplus.2026.100200 (PMC13316760; doi:10.1016/j.mbplus.2026.100200)
Supplement: Supplementary file 5 — Supplementary figure legends and Supplementary Tables 1 and 2. Legends for Supplementary Figures 1–4, collagen fibril diameter distribution, and primer sequences used for quantitative PCR. [file mmc5.docx]

**Supplementary Figure 1. Validation of Piezo1 recombination in Scx-lineage tendons.**

(**A**) Genotyping PCR of genomic DNA from Achilles tendon and adjacent muscle in Control and *Piezo1* *^p-t-ko^* female mice after tamoxifen induction. PCR was performed using three primers to distinguish wild-type, floxed, and recombined alleles. The wild-type allele is detected at ~160 bp, the floxed allele at ~330 bp, and the recombined knockout allele at ~230 bp. The non-recombined alleles (wild-type or floxed) are detected in both control and knockout samples. The recombined band is observed specifically in tendon samples from *Piezo1* *^p-t-ko^* female mice, but not in muscle, confirming tissue-specific recombination. **(B)** Representative immunostaining images for PIEZO1 in Achilles tendon sections from Control and *Piezo1 ^p-t-ko^* mice at the analyzed age. Green, PIEZO1; blue, DAPI. Scale bar, 100 μm. **(C)** Quantitative analysis of PIEZO1 immunostaining intensity in Achilles tendon midsubstance regions. Data are presented as mean ± SD. n = 5 mice per group.

**Supplementary Figure 2. Matrisome-focused transcriptomic analysis of Control and *Piezo1* *^p-t-ko^* female tendons.**

(**A**) Volcano plot showing differentially expressed matrisome genes. Representative downregulated genes in *Piezo1* *^p-t-ko^* tendons include Col1a1, Dcn, Fmod, Comp, Col3a1, and Thbs4. (**B**) Heatmap of the top 20 downregulated and top 20 upregulated matrisome genes, showing clear clustering of Control and *Piezo1* *^p-t-ko^* female samples.

**Supplementary Figure. 3. Transcriptomic changes in mechanotransduction-related pathways following Piezo1 deletion.**

(A) Gene set enrichment analysis (GSEA) of GO biological processes and MSigDB hallmark gene sets. Mechanotransduction-related processes, including actin-mediated cell contraction, actin filament–based movement, and calcium ion transport, were enriched. Mitochondrial and oxidative phosphorylation pathways were also enriched. Bar plots show normalized enrichment scores (NES), colored by −log10(FDR). (B) Expression changes of selected mechanotransduction-related genes. Dot color represents −log10(p-value), and filled symbols indicate statistically significant changes (FDR < 0.05).

**Supplementary Figure 4. Tendon-specific Piezo1 deletion does not alter adjacent muscle morphology or gene expression.**

(A) Weights of gastrocnemius (GA), tibialis anterior (TA) and rectus femoris (RF) muscles in Control and *Piezo1* *^p-t-ko^* female mice at 18 weeks (n = 6 per group). Data are mean ± SD; n.s., not significant by unpaired Student’s t-test. (B) RNA-seq comparison of muscle transcriptomes from Control and *Piezo1* *^p-t-ko^* female mice (n = 4 per group). **Supplementary Table 1. Collagen fibril diameter distribution**

| Genotype | Mice (n) | Fibrils analyzed | Mean (nm) | SD | Variance | Q1 | Median | Q3 |
| --- | --- | --- | --- | --- | --- | --- | --- | --- |
| Control | 6 | 900 | 134 | 43.5 | 1890 | 104 | 140 | 165 |
| *Piezo1 ^p-t-ko^* | 6 | 900 | 119 | 50.0 | 2503 | 77.9 | 115 | 156 |

Data represent collagen fibril diameter measurements obtained from transmission electron microscopy images of Achilles tendons. Approximately 150 fibrils were measured per tendon from six mice per genotype. SD, standard deviation; Q1, first quartile (25th percentile); Q3, third quartile (75th percentile).

**Supplementary Table 2. Primer sequences used for quantitative PCR**

| Gene | Forward primer (5'–3') | Reverse primer (5'–3') |
| --- | --- | --- |
| *Gapdh* | TTGTGGAAGGGCTCATGACC | TCTTCTGGGTGGCAGTGATG |
| *Piezo1* | CTCACAGACAGGTGTTCATC | GCAAACTCACGTCAAGGAGA |
| *Mkx* | GATGGCGACTCCTGCTCTGA | CGGTCTGCCGCCAGCTTTTA |
| *Scx* | CCTTCTGCCTCAGCAACCAG | GGTCCAAAGTGGGGCTCTCCGTGACT |
| *Dcn* | CTATGTGCCCCCTACCGATGC | CAGAACACTGCACCACTCGAAG |
| *Fmod* | CAGCTGCAGAAGATCCCTCC | AGTTCATGACGTCCACCACC |
| *Col1a1* | GAGCGGAGAGTACTGGATCG | GCTTCTTTTCCTTGGGGGTTC |
